# Inhibitors of the Cancer Target Ribonucleotide Reductase, Past and Present

**DOI:** 10.3390/biom12060815

**Published:** 2022-06-10

**Authors:** Sarah E. Huff, Jordan M. Winter, Chris G. Dealwis

**Affiliations:** 1 Department of Pediatrics, University of California, San Diego, CA 92093, USA; sahuff@health.ucsd.edu; 2 Department of Surgery, Division of Surgical Oncology, University Hospitals Cleveland Medical Center, Akron, OH 44106, USA; jordan.winter@uhhospitals.org; 3 Department of Pharmacology, Case Western Reserve University, Cleveland, OH 44106, USA; 4 Department of Chemistry, Case Western Reserve University, Cleveland, OH 44106, USA

**Keywords:** ribonucleotide reductase, ribonucleotide reductase inhibitors, cancer chemotherapy, small molecule, drug discovery, hydrazone, acyl hydrazone, gemcitabine, fludarabine, clofarabine, cladribine, triapine, COH 29, pancreatic cancer, breast cancer, small cell lung cancer

## Abstract

Ribonucleotide reductase (RR) is an essential multi-subunit enzyme found in all living organisms; it catalyzes the rate-limiting step in dNTP synthesis, namely, the conversion of ribonucleoside diphosphates to deoxyribonucleoside diphosphates. As expression levels of human RR (hRR) are high during cell replication, hRR has long been considered an attractive drug target for a range of proliferative diseases, including cancer. While there are many excellent reviews regarding the structure, function, and clinical importance of hRR, recent years have seen an increase in novel approaches to inhibiting hRR that merit an updated discussion of the existing inhibitors and strategies to target this enzyme. In this review, we discuss the mechanisms and clinical applications of classic nucleoside analog inhibitors of hRRM1 (large catalytic subunit), including gemcitabine and clofarabine, as well as inhibitors of the hRRM2 (free radical housing small subunit), including triapine and hydroxyurea. Additionally, we discuss novel approaches to targeting RR and the discovery of new classes of hRR inhibitors.

## 1. Introduction

Ribonucleotide reductase (RR) is an essential enzyme found in all living organisms; it catalyzes the conversion of ribonucleoside diphosphates (RNDPS) to deoxyribonucleoside diphosphates (DNDPS) [1,2]. As this is the rate-limiting step in dNTP synthesis, human ribonucleotide reductase (hRR) is crucial for maintaining a balanced nucleotide pool within the cell and is integral to DNA synthesis [1,2,3]. During the S-phase of the cell cycle, hRR expression is elevated, making hRR an attractive drug target for proliferative diseases, including cancer [4,5,6]. The majority of cancer drugs targeting hRR are nucleoside analogs that mimic the chemical structure of natural substrates [7,8,9,10,11]. The most successful of these antimetabolites is gemcitabine, which is one of the very few FDA-approved clinical treatments for pancreatic cancers [12,13]. However, antimetabolites such as gemcitabine can inhibit numerous off-target enzymes involved in DNA synthesis and repair, leading to DNA strand breaks and apoptosis [10,14,15,16]. Additionally, gemcitabine confers high rates of therapeutic resistance, where only ~10% of pancreatic cancer patients will respond to single-agent gemcitabine treatment [17]. To overcome these limitations, there were several attempts to identify non-nucleoside inhibitors of hRR in recent years, many of which were designed to inhibit hRR either by targeting the hRRM2 (β subunit) directly or by manipulating its tightly controlled oligomeric regulation. Although there are several excellent reviews on hRR as a cancer drug target [18,19], there have been significant advances in the discovery of inhibitors of hRR since the most recent reviews focused on this subject [4,20], and thus, an update is required. In this review, we discuss the mechanism, activity, and clinical applications of the classic antimetabolite inhibitors clofarabine and gemcitabine, as well as the broad class of inhibitors targeting the small subunit, including triapine and hydroxyurea. Additionally, we discuss novel strategies to target RR, including the discovery of modulators of the hRRM1 (α) oligomeric state, the acylhydrazone class of hRRM1 inhibitors (including the C-site inhibitor NSAH), a high throughput PCR assay method to identify inhibitors of *Pseudomonas aeruginosa* (*P.aeruginosa*) RR, and iron chelators as transmetalative inhibitors of hRRM2.

## 2. Ribonucleotide Reductase

Ribonucleotide reductase was first characterized in 1961 by the Reichard group at the Karolinska Institute in Stockholm, Sweden. The enzyme is composed of two subunits, namely, RR1 and RR2 [1,2]. The large RR1 subunit (also known as the α subunit) contains the catalytic site (C-site) and all allosteric sites, while the small RR2 subunit (also known as the β subunit) contains the diferric-tyrosyl (Y√) radical cofactor that initiates nucleotide reduction via a putative long-range proton-coupled electron transfer (PCET) pathway approximately 35 Å away [1]. Recently, a new form of RR2 was discovered named p53R2 that operates outside of the S-phase of the cell cycle to maintain sufficient levels of dNTP pools for DNA repair. P53R2 is [21,22] expressed in quiescent and post-mitotic cells, where it combines with RR1 to synthesize the dNTPs required for nuclear DNA repair and mitochondrial DNA replication [21]. RRs are divided into three classes by the metal cofactors that generate their free radical electron. In this review, we were interested primarily in hRR, which is a class I enzyme and uses a tyrosyl free radical for catalysis. The class I enzymes are further divided into five subcategories Ia, Ib, Ic, Id, and Ie based on the different radical cofactors used in catalysis [23,24,25,26,27]. Below, we discuss the mechanism of class Ia enzymes, which include human, *Escherichia coli* (*E. coli*), and *Saccharomyces* cerevisiae (*S. cerevisiae*) RRs.

### 2.1. The Structure and Allosteric Regulation of Ribonucleotide Reductase

**Figure 1:**
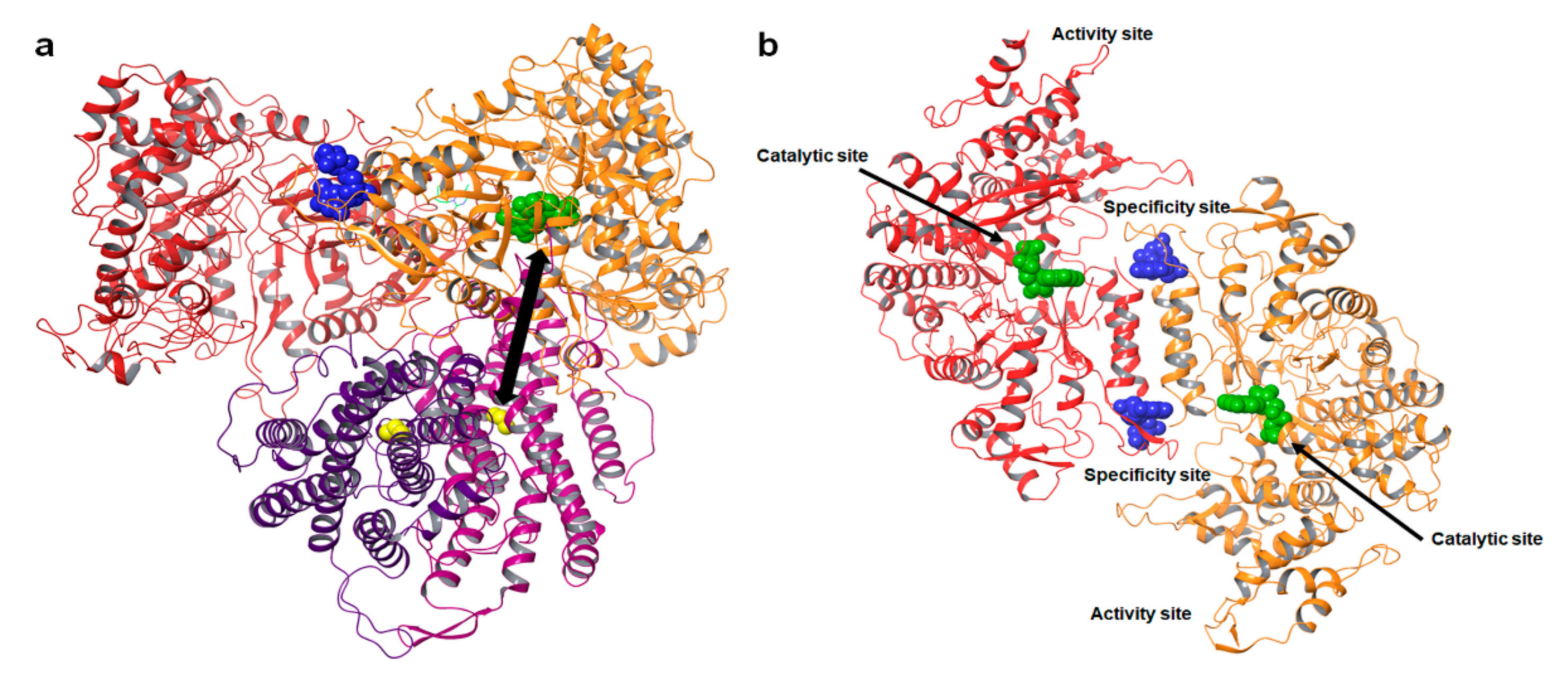
Structure of ribonucleotide reductase. (**a**) Model of the *E. coli* α_2_β_2_ dimer complex (PDB ID: 6W4X) [32]. The α dimer is shown in red and orange. The nucleotide bound at the C—site is represented as green spheres. The nucleotide effect abound at the S—site is represented as blue spheres. The β subunit is shown in magenta and purple; the yellow spheres represent the radical generating di-iron cluster. The iron cluster and C-site are separated by a distance of over 35 Å, shown as a double arrow. (**b**) Structure of the hRRM1 dimer. The nucleotide bound at the catalytic site (C-site) is rendered as green spheres. The specificity site (S-site) controls substrate selection at the C-site and is represented as blue spheres. The activity site (A-site) controls the overall activity of the enzyme by binding the natural allosteric activator ATP or inhibitor dATP.

The active form of eukaryotic RR consists of dimeric or multimeric forms of the RR1 (α) and RR2 (β) subunits, such as α_2_β_2_ and α*_6_*β*_N_* (where *n* = 2, 4, or 6) [28,29,30,31]. Catalysis occurs in the RR1 (α) subunit, which contains the catalytic site (C-site) and two additional allosteric sites, namely, the specificity site (S-site) and the activity site (A-site) (Figure 1).

**Figure 2:**
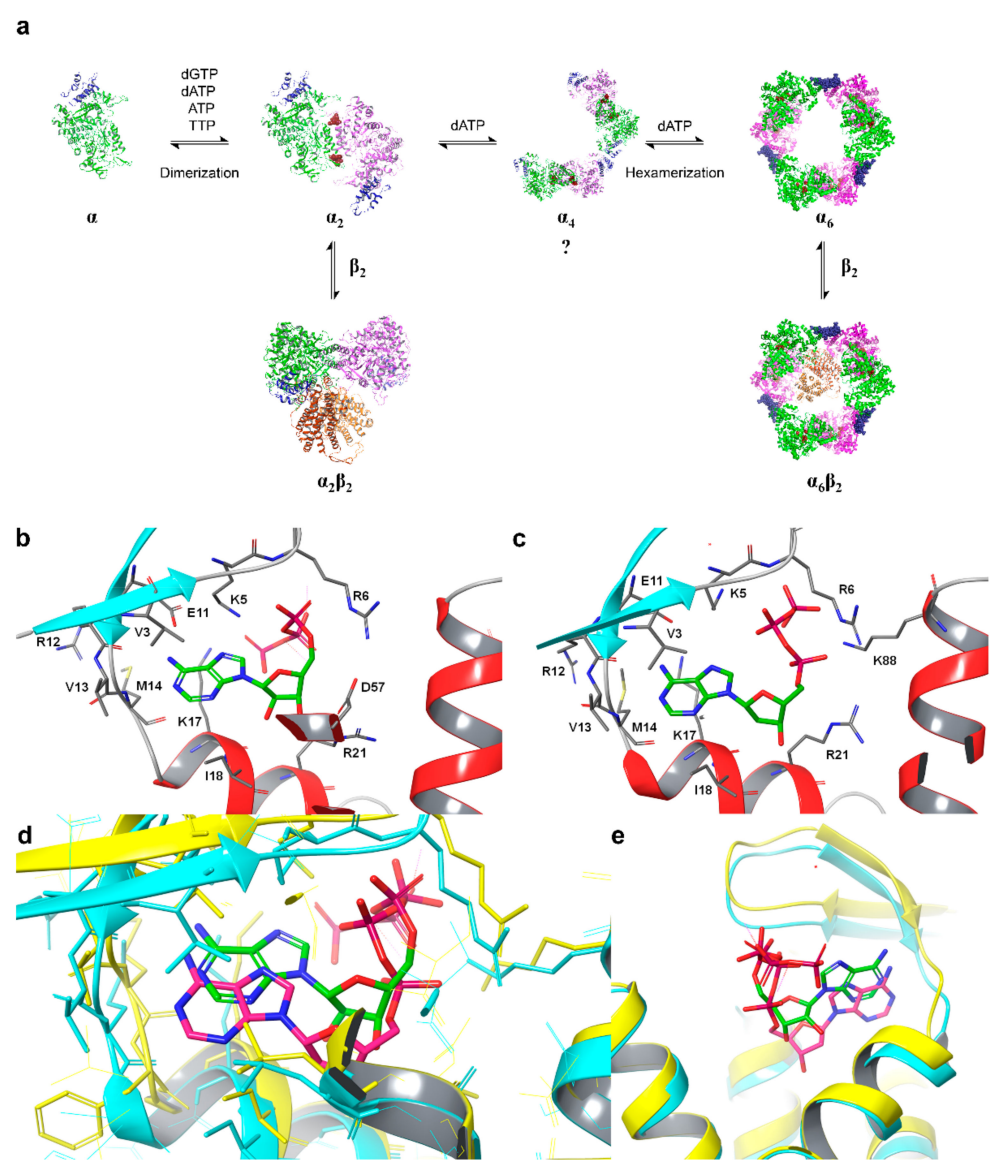
(**a**) Model for dATP-dependent oligomerization of eukaryotic RRs. The binding of effectors to the S-site causes dimerization and the binding of dATP to the A-site causes the formation of hexamers via a hypothesized short-lived tetramer intermediate or the immediate association of three dimers to form a hexamer (question mark). Effectors bound at the S-site are maroon spheres, and the A-site is rendered as a blue ribbon. (**b**) ATP-hRRM1 complex. The main chain is rendered in gray (helices—red, strands—cyan), while ATP is shown in elemental green. (**c**) dATP-hRRM1 complex. The main chain is rendered in gray (helices—red, strands—blue), while dATP is shown in elemental green. (**d**) Superposition of A and B. The ATP-hRRM1 complex is rendered in yellow ribbon with ATP in elemental green. The dATP-hRRM1 complex is rendered as a cyan ribbon with dATP in elemental magenta. (**e**) The ATP-binding cones of hRRM1 in complex with TTP and ATP (yellow) or TTP and dATP (cyan). ATP is shown in elemental green and dATP in elemental magenta (dTTP is not shown).

The A-site controls the overall activity of the enzyme by binding either ATP or dATP, while the S-site controls which of the four ribonucleoside diphosphate substrates bind to the C-site [2]. A model for the oligomeric equilibrium of hRRM1 and its control via allosteric effector binding to the S-site and A-site is presented in Figure 2a. Crystal structures of both ATP, i.e., the natural activator, and dATP, i.e., the natural inhibitor, in complex with the A-site of hRRM1 were reported by the Dealwis group in 2011 (Figure 2b–e) [30]. ATP was observed to bind deep within a pocket at the tip of the N-terminal domain, which is an area also referred to as the ATP-cone [30]. The natural inhibitor dATP was found to bind the A-site with higher affinity compared with the activator ATP, primarily due to differences in interactions with Ile 18. X-ray crystallography and cryo-EM studies demonstrated that the α_6_ hexamer consists of hRRM1 trimer of dimers [30,33]. Although there are dATP-bound α_6_ and α_6_β_2_ structures, so far, there are no structures for the ATP hexamer [30,33]. Nevertheless, mutagenesis analysis demonstrated that the active ATP-bound α_6_ structure is likely to be different from the dATP-inhibited hexamer, possibly explaining the differing activities. The dATP-bound hexamer structures reveal that inhibition occurs due to the positioning of β, which is too far away to transfer the free radical to the α active site.

For more than four decades, investigators in the RR field have been fascinated by how this enzyme is able to recognize the four nucleotide diphosphates and convert them to their respective deoxy forms that provide the building blocks of life [2]. Most of the model studies with respect to molecular recognition were conducted using the perennial *E. coli* model of an α_2_β_2_ holo-complex. Insights into the molecular basis of the substrate recognition of RR came to light with the availability of crystal structures with their cognate effector–substrate complexes from *E. coli*, *Thermotoga maritima* (*T. maritima*), *S. cerevisiae*, and human RR, which are described in detail below [30,34,35,36]. Based on biochemical and structural data, a simple model emerged for maintaining balanced nucleotide pools that require elegant effector-based substrate recognition by the RR enzyme. Substrate recognition involves crosstalk between the S- and C-sites that are located in adjacent monomers composing the dimeric α_2_ molecule (see Figure 1b). The S-site is located >15 Å from the C-site and influences substrate binding by inducing subtle changes to the flexible loop 2 region (residues 285–295, hRRM1 numbering) of the C-site, which promote the binding of a specific substrate [30,31,35,37]. Substrate recognition is controlled by four allosteric effectors, namely, dGTP, dTTP, ATP, and dATP, binding at the S-site. In 1969, Brown and Reichard showed that this regulation is tightly controlled by the concentration of deoxynucleotides in the cell [1,2]. As the concentration of dNTPs decreases, ATP that has an almost constant concentration of 3–5 mM will bind at the A-site, activating RR. ATP will also bind to the S-site, which promotes the binding of CDP and UDP, which are then converted to dCDP and dUDP by RR. Phosphorylation of dCDP gives dCTP. dUDP is reduced to dUMP prior to conversion to dTMP by thymidylate synthase, which leads to an increase in dTTP concentrations [38]. As the concentration of dCTP and dUTP increases, dTTP outcompetes ATP at the S-site, promoting the catalysis of GDP to dGDP. As the concentration of dGTP increases, it will bind the S-site to promote the synthesis of dADP, which then increases the concentration of dATP in the cell. At this point, dATP will bind to the A-site, inhibiting the enzyme. As dATP has a higher binding affinity for the A-site than ATP, RR is only activated when the cellular concentration of dATP is low.

Allosteric effectors that bind to the S-site control substrate binding at the C-site by altering the conformation of a polypeptide chain known as loop 2, located within the C-site [30,35,36,39]. The molecular basis of recognition between effector–substrate pairs was established primarily through X-ray crystallography and site-directed mutagenesis, beginning with the first crystal structure of the substrate/effector pair GDP and dTTP in complex with RR1 by Eriksson et al.in *E.Coli* in 1997 [34]. Several labs demonstrated that subtle changes in the conformation of loop 2 will preferentially accommodate the binding of one substrate (or two, in the case of ATP) at the C-site [30,35,36,37]. This preference is largely based on steric constraints. The loop 2 residue Arg 293 is observed to form strong π–π stacking and/or π–cation interactions with the nucleoside base of the purines. In *E. coli* and *T. maritima*, Arg 293 forms a salt bridge with the phosphate groups of substrates bound in the C-site [35,36]. When purine substrates are bound, the loop 2 residue Gln 288 is observed to swing away from the C-site to accommodate the binding of the larger nucleoside bases. However, the CDP-RR1 complex shows that when smaller pyrimidine substrates bind, Gln 288 will shift further into the C-site. R293A mutants in yeast showed a loss of activity for both ADP and CDP substrates, further indicating that this residue is critical for substrate recognition and RR activity [39].

### 2.2. The Catalytic Mechanism of Class Ia RRs

**Scheme 1:**
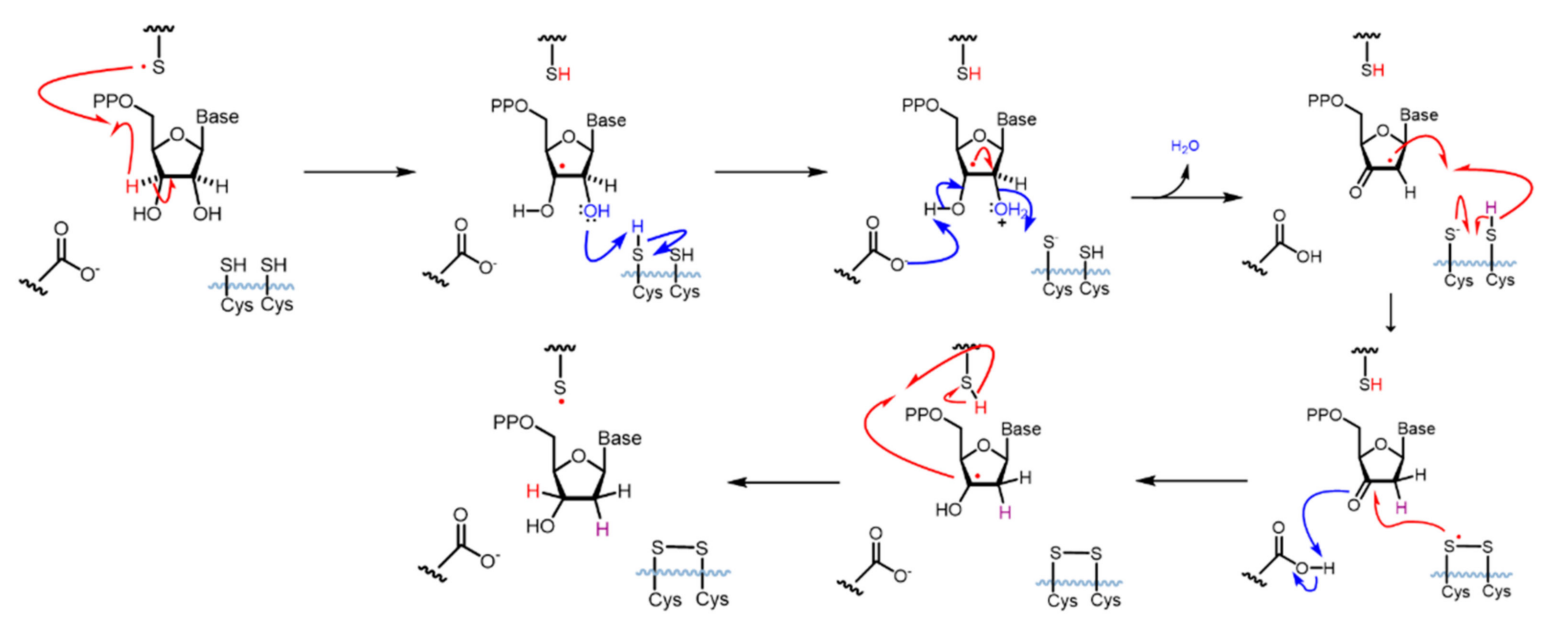
Catalytic mechanism of hRR. Radical movement is shown with red arrows. The movement of electron pairs is shown with blue arrows. Residues are numbered according to human RR.

The RR2 (β) subunit contains a stable diferric-*tyrosyl (Y√) radical cofactor*, which is required for catalysis [40]. Prior to catalysis, radical hopping occurs through stable aromatic residues from the diferric-tyrosyl in the RR2 (β) subunit 30–35 Å ito a catalytic cysteine in the C-site by a proton-coupled electron transport (PCET) mechanism [23,40,41,42,43,44,45]. This initiates the conversion of the ribonucleotide to its deoxy form through the mechanism illustrated in Scheme 1, where all residue numbering refers to the human system [42,43,46]. The radical is transferred to the C3′ position of the ribose ring of the substrate, generating a 3′-nucleotide radical. Glutamate 431 (*E. coli* 441) facilitates radical movement to the C2′ position by functioning as a general base catalyst for deprotonation of the 3′-OH. C443 (*E. coli* 462) facilitates the removal of the C2′ hydroxy group as a water molecule in a rapid, irreversible step. The subsequently formed 3′-keto-2′ radical is reduced by the PCET mechanism to generate the 3′-ketodeoxynucleotide and the disulfide radical anion (C443–C218 human; C462–C225 *E. coli*). The disulfide radical anion reduces the 3′-ketone with PCET, where the H atom is donated by E431 (*E. coli* 441). Finally, the H-atom removed from the C3′ ribose ring is returned to form the dNDP product and the radical is then transferred back to C429 (*E. coli* 439) and returns to the iron center in RR2 via the PCET pathway [40]. Before additional catalysis can occur, the disulfide bond between C443 and C218 must be reduced. This reduction is dependent upon the thioredoxin and glutaredoxin system, which, in turn, is reduced by thioredoxin reductase and glutathione [47,48]. Both systems are ultimately reduced by NADPH. This cycle of PCET and C-site cysteine reduction is required for each catalytic turnover. The recently reported cryo-EM structure of a trapped radical *E. coli* holo-complex provided the first glimpses of a structural map of this catalytic mechanism [32].

## 3. Ribonucleotide Reductase as a Target for Cancer Therapeutics

### 3.1. The Role of RR in Cancer Biology

The most widely recognized hallmark of cancers is uncontrolled proliferation, which requires a sufficient supply of dNTPs. As hRR plays a crucial role in maintaining dNTP pools, the role of hRR in cancer proliferation has been of scientific interest for decades. In the 1970s, studies of rat hepatomas determined that RR activity is highly correlated with tumor growth rate, where enzymatic activity was approximately 200-fold higher in fast-growing tumors than in slow-growing tumors [49]. Either the hRRM1 or hRRM2 subunits are known to be overexpressed in a wide variety of cancers, including gastric, ovarian, colorectal, brain, breast, liver, and non-small-cell lung (NSCLC) cancers [50,51,52,53,54,55,56,57]. Overexpression of p53R2 is also observed in melanoma, oral carcinoma, esophageal, and NSCLC. In breast and epithelial ovarian cancers, the overexpression of p53R2 is correlated with the tumor grade, and this subunit is also highly expressed in malignant melanoma relative to benign skin lesions [58,59,60]. An analysis of the ONCOMINE dataset identified that the hRRM1 subunit is among the top 10% of most overexpressed genes in 30 of the 170 studies examined, including the brain, central nervous system, lung, and sarcoma datasets [4].

The effects of hRRM1 or hRRM2 overexpression are complex and varied. Dysregulation of hRR results in imbalanced nucleotide pools, which can inhibit DNA synthesis and replication and interfere with DNA damage repair [3]. While elevated dNTP levels can promote mutagenesis by DNA insertion and the escape of DNA polymerase proofreading via the “next-nucleotide effect” or increased frameshift mutations, enhanced hRR activity can also support DNA repair mechanisms. In cancers, these DNA repair mechanisms can be employed to protect against DNA damaging agents, such as the platinum class of chemotherapies. Overexpression of hRRM2 has been associated with increased invasion in human carcinoma cells and increased VEGF production, suggesting that hRRM2 can promote tumor invasion and angiogenesis [60]. Like hRRM1, elevated expression of hRRM2 can confer resistance to genotoxic chemotherapies, such as 5-fluorouracil, platinum drugs, or radiation therapy [61,62,63,64,65,66,67,68,69,70]. Overexpression of either subunit is also predictive of a poor response to hRR inhibitors, such as gemcitabine or triapine [71,72,73,74,75,76]. 

As the expression of p53R2 can be induced by the tumor suppressor p53 and is known to promote p21 accumulation and G1 arrest, it was originally expected that p53R2 would also play a tumor suppressor role [22]. However, overexpression of p53R2 is found in many cancers and knockdown studies have identified that p53R2 suppression prevents proliferation [77]. Furthermore, hRRM2 and p53R2 were shown to promote NSCLC in vivo, although this has not been replicated for other cancers [78]. The mechanism by which the hRRM2 and p53R2 subunits promote proliferation in cancers needs further examination, although there are several possibilities. Elevated hRRM2 could promote mutagenesis by altering dNTP pools such that base misinsertions, insertion–deletion events, and uracil misincorporation occur at a higher frequency. Several studies have also indicated hRR-driven dNTP alterations impact senescence and apoptosis pathways. hRRM2 and p53R2 overexpression were also linked with increased G→T transversions, an indicator of oxidative DNA damage [78]. As the Y (radical) housed in the hRRM2 subunit is transient and highly reactive, it is possible that overexpression of hRRM2 could result in the generation of reactive oxygen species (ROS) [79,80]. However, it has not yet been demonstrated that hRRM2 is capable of generating such ROS. 

While there remains much to be learned about the complex mechanism of hRR’s promotion of cancer proliferation, its role in maintaining dNTP homeostasis has made it an attractive therapeutic target for decades.

### 3.2. Cancer Chemotherapeutics Targeting hRRs

Inhibiting hRR can lead to mutations, causing the incorporation of incorrect nucleotides, as well as chromosomal instability through replication fork collapses, double-strand breaks, and shortening of telomeres that cause chromosomal fusions [81,82]. Numerous small molecule inhibitors have been developed to inhibit hRR through a variety of mechanisms. The most important anticancer inhibitors can be divided into two classes, those that target the hRRM1 subunit, such as gemcitabine and clofarabine, and those that target the hRRM2 subunit, such as hydroxyurea [20]. Drugs targeting the hRRM1 subunit are primarily antimetabolites, which mimic natural substrates of hRR [7,8,9,10,11,15,18,83,84,85,86]. These drugs are administered in their nucleoside forms in order to be membrane permeable; however, in the cytoplasm, they are phosphorylated by cellular kinases to their mono, di, and triphosphate forms [87]. hRRM2 inhibitors compromise the integrity of the iron-tyrosyl radical center by either scavenging the radical electron, such as with hydroxyurea, or chelating iron, such as with triapine [88,89,90,91,92]. Additionally, recent studies have identified small molecule inhibitors that disrupt the binding of the hRRM1 and hRRM2 subunits. In this review, we focus on the four most clinically important nucleoside analogs, namely, cladribine, fludarabine, clofarabine, and gemcitabine, and provide a broader discussion of attempts to discover and develop non-nucleoside inhibitors of hRR.

## 4. The Antimetabolite Class of hRRM1 Inhibitors

The majority of clinically used drugs targeting hRR are nucleoside analogs that bind to the C-site or both C- and A-sites of the hRRM1 subunit, and the early development is described in references [86,93]. Among these are fludarabine, cladribine, gemcitabine, and clofarabine [7,8,9,10,11,15,18,83,84,85,86,93]. These four drugs impair DNA synthesis and repair pathways in part through the inhibition of RR, although most are also known to inhibit a variety of off-targets, including DNA polymerase, thymidylate synthase, and both cytidine deaminase and CTP synthase in the case of gemcitabine [10,14,15]. Although hRR is inhibited by these drugs, the major toxicities derive from DNA chain termination. Table 1 contains the structures, mechanisms, and indications of FDA-approved nucleoside analog inhibitors of hRR, as well as the radical scavenger hydroxyurea. The structures and mechanisms of all remaining hRR inhibitors are presented in Table 2 and Table 3.

### 4.1. The Antimetabolite Class of RR Inhibitors: Cladribine (CLA) and Fludarabine (FLU)

Fludarabine is approved for the treatment of various leukemias and lymphoma, including chronic lymphocytic leukemia, acute myeloid leukemia, acute lymphocytic leukemia, and non-Hodgkin’s lymphoma [94,95,96,97]. Fludarabine is a structural mimic of ADP with the addition of a fluorine atom on the 2 position of the adenine base and stereochemical inversion of the C2′ hydroxyl of the ribose ring. Typically sold as a monophosphate (Fludara), fludarabine can become further phosphorylated intracellularly to the diphosphate and triphosphate forms, the latter of which is known to inhibit hRR [95], DNA polymerase [98], DNA ligase [99], and DNA primase [100]. Fludarabine is commonly administered in combination with other chemotherapies, such as cyclophosphamide, mitoxantrone, dexamethasone, and rituximab, for the treatment of non-Hodgkin’s lymphomas or as part of the FLAG or FLAMSA treatment regimens for AML, where it is administered with cytarabine and granulocyte colony-stimulating factor. Cladribine is approved for first- and second-line treatment of hairy cell leukemia and for B-cell chronic lymphocytic leukemia [101,102]. Being a structural analog of adenosine, cladribine contains a chlorine atom at the 2 position, which renders the compound partially resistant to breakdown by adenosine deaminase, causing it to accumulate intracellularly and interfere in DNA synthesis and repair. Like other nucleoside analogs, it is phosphorylated intracellularly by deoxycytidine kinase to produce both the diphosphate and triphosphate forms. Incorporation of the triphosphate forms of fludarabine and cladribine into DNA strands by DNA polymerase leads to increased DNA strand breaks and the activation of transcription factor p53, eventually culminating in apoptosis [103,104]. The cellular efficacy of cladribine is dependent upon the ratio of the active triphosphate and the 5′-nucleotidase (5′-NT) family of enzymes, which dephosphorylate cladribine into the inactive prodrug form. As this ratio is highest in T and B lymphocytes, they are particularly susceptible to cladribine-mediated apoptosis, while other non-hematological malignancies are not significantly affected. 

The mechanism of inhibition of hRR by fludarabine and cladribine was determined in part via a comparison of their effects on α_6_ hexamer formation in human cell lines [105]. ClA and FlU nucleotides were determined to promote the formation of α_6_ hexamers, although the hexamer formed in the presence of FlUDP was only observed in gel filtration studies if FlUDP was supplemented in the running buffer; this unexpected result was not observed for other nucleotides, including FlUTP, and suggests that the hexameric states formed by the diphosphate and triphosphate forms are structurally distinct and maintained post-ligand dissociation. This theory is supported by the additional finding that trypsin protease cleavage of the hexamers induced by different nucleotide inhibitors occurred at different rates. While both FlUDP and ClADP are known to interact with the C-site of hRRM1, this interaction appears to be less relevant than the hexamer-inducing interactions, as inhibition of hRR is dependent upon the ability of the enzyme to form α_6_ hexamers. The D57N-α mutant was uninhibited by either ClA or FlU because the D57N mutant is unable to form hexamers, which are required for inhibition [105].

### 4.2. The Antimetabolite Class of hRR Inhibitors: Clofarabine

Clofarabine was approved in 2004 for the treatment of relapsed or refractory acute lymphoblastic leukemia in children [106,107,108]. This drug is a mimic of adenine diphosphate (ADP), with the critical change of the C2′ ribose hydroxyl to a fluorine atom and there is a chlorine substitution at the 2 position of the adenine ring. As the side effects of clofarabine are severe (hematologic toxicity, febrile neutropenia, hepatobiliary toxicity, renal toxicity) and can lead to systemic inflammatory response syndrome, which can cause multiple organ failure, the drug is approved only for patients for whom two other treatment methods have failed [109]. Clofarabine can exist in the monophosphate, diphosphate, and triphosphate forms, with the triphosphate form heavily favored in cells (ratio of ClFDP:ClFTP up to 1:7) [108]. While the diphosphate form binds to the catalytic site to inhibit hRR reversibly in a time-dependent manner, the triphosphate form will inhibit both the A-site of hRR and DNA polymerases α and ε [83]. In contrast to the diphosphate form, ClFTP inhibition of hRR is time independent. Both forms were shown to promote the formation of α hexamers [83,110]. Interestingly, in contrast to the dATP-α_6_ complex, dissociation of either ClFDP or ClFTP leaves the α subunit trapped in an inactive α_6_ hexamer conformation. Site-directed mutagenesis studies identified A-site residue D57 to be essential for the formation of this inactive hexamer conformation. The clofarabine triphosphate-induced inactive hexamer structure from mammalian cells was determined using cryo-EM [110]. The conclusion from this study was that by using cell culture, the authors were able to induce hexamer structures of the α subunit in sufficient quantities to be able to capture the cryo-EM image; however, the β subunit appeared to be disordered and could not be assigned in the structure. Clofarabine triphosphate induces apoptosis by incorporating into DNA strands, causing breaks within the strand. The triphosphate form was also shown to directly influence mitochondrial integrity by disrupting the mitochondrial membrane, which leads to apoptosis [111,112].

### 4.3. The Antimetabolite Class of hRR Inhibitors: Gemcitabine

**Figure 3:**
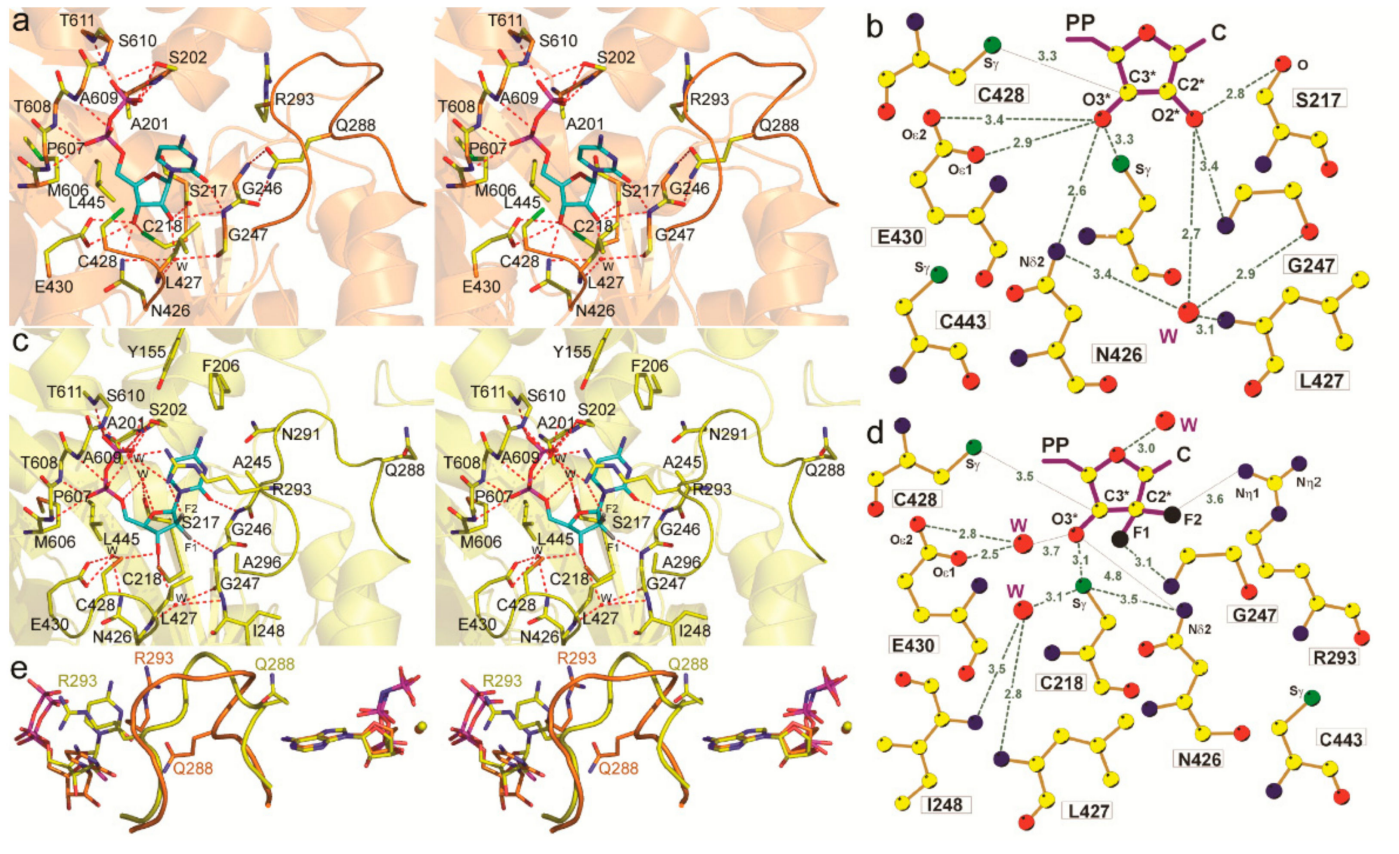
Crystal structure of ScRR1 in complex with gemcitabine. (**a**) Stereo view of CDP (orange). Interacting atoms: oxygen, red; nitrogen, blue; phosphate, magenta; sulfur, green; substrate carbons, cyan; protein non-Cα carbons, yellow; Cα carbons, as secondary structure, orange. (**b**) Stereo view of gemcitabine diphosphate. Interacting atoms are colored as in (**a**), except that sulfur is orange; Cα carbons, as secondary structure, are yellow; and fluorines are gray. (**c**) Ligand plot of CDP ribose interactions. Colors are as in (**a**), except that carbons are yellow. (**d**) Ligand plot of gemcitabine diphosphate interactions. The van der Waals contact with L427 is omitted for clarity. (**e**) Stereo view of the loop 2 superposition of AMPPNP–CDP (orange) and AMPPNP–gemcitabine diphosphate (yellow). Substrate/inhibitor is seen on the left, and the effector is on the right. The color scheme is the same as in (**c**), but fluorine is black. Figure reprinted with permission from Xu et al. [118].

Gemcitabine is a unique nucleoside drug. Unlike other nucleoside drugs, gemcitabine is efficacious against solid tumors, as well as hematological malignancies. Gemcitabine is approved as a front-line treatment for pancreatic cancer after patients are first treated with a combination of surgical resection and folfiinox [113]. In fact, it is the most commonly used drug against pancreatic cancer. In metastatic non-small-cell lung cancer and metastatic bladder cancer, gemcitabine is combined with cisplatin [114,115]. Gemcitabine is also used as a second-line treatment for ovarian cancer (in combination with carboplatin) [116] and metastatic breast cancer (in combination with paclitaxel) [117]. It is a structural mimic of CDP with the substitution of two fluorine atoms at the C2 position of the ribose ring. Crystal structures of gemcitabine demonstrate that it binds at the catalytic site of the α subunit in an altered conformation to the natural substrate CDP (Figure 3) [118]. The mechanism of inhibition by gemcitabine has been investigated both in the presence and absence of reducing agents (thioredoxin, DTT) [119,120].

In either environment, inhibition is time dependent and irreversible with full hRR inactivation and binds with a stoichiometry of 0.5 equivalent gemcitabine per equivalent hRRM1. When bound at the catalytic site of hRR, the radical is transferred to the C3′ position of the ribose ring but is unable to transfer to the C2′ position as the geminal fluorine atoms cannot react as the hydroxyl group of the natural substrate. At this stage, the ribose ring breaks apart, and in a reducing environment, may alkylate the enzyme, inhibiting it irreversibly [119]. This alkylation was confirmed in *Lactobacillus leichmannii (L. leichmannii)*, where mass spectrometry identified the alkylation of C419 [119,120]. However, this alkylation has not yet been observed in the human enzyme, and gemcitabine’s irreversibility may come from the quenching of the transient thiol radical, which was observed in the absence of reductants [119,120]. This radical scavenging most likely occurs when the enzyme is in its oxidized state. In addition to inhibiting hRR, gemcitabine is phosphorylated in the cell and can replace dCTP in DNA replication, leading to apoptosis [10,11,121]. As the diphosphate form inhibits hRR and depletes the concentration of natural dNTPs in the cell, the levels of dCTP reduce. As dCTP is a natural feedback inhibitor of deoxycytidine kinase, dNTP depletion leads to the enhanced production of gemcitabine triphosphate such that it can compete for incorporation into DNA strands. In this way, gemcitabine’s cytotoxicity is self-potentiating [10,87,122]. Additional studies are needed to fully characterize the effects of this self-potentiating mechanism of cytotoxicity. Other side targets of gemcitabine include thymidylate synthase, cytidine deaminase, deoxycytidine kinase, and DNA polymerase [10,11,15,16,85]. 

Clinical use of gemcitabine is further limited by the propensity for tumors to develop a resistance to it [123,124,125,126,127]. While this resistance could be attributed to many pathways, gemcitabine-induced upregulation of anti-apoptotic proteins, such as BEX2, Bcl2A1, and CXCR4, could be a major factor [124,128,129]. Increased expression levels of several multidrug-resistant (MDR) genes, such as ABCC1, ABCC3, ABCC5, and ABCB1, in pancreatic cancer cells also promote drug efflux, lowering the concentration of gemcitabine within the tumor over time [130,131,132,133,134,135]. 

While gemcitabine remains a mainstay of pancreatic cancer chemotherapy, only approximately 10% of patients will respond to gemcitabine treatment. Those that do respond can expect to survive an additional 6–8 weeks after starting treatment, with a 5-year survival rate of only 7% [130]. In the past two decades, other than adjuvant chemotherapy treatment, such as folfirinox, and the combination of nab-paclitaxel with gemcitabine, only one additional drug has been approved to treat pancreatic cancer, namely, the epidermal growth factor receptor (EGFR) inhibitor erlotinib [136,137,138,139,140,141,142,143]. Erlotinib is approved as a combination therapy with gemcitabine for certain local pancreatic tumors and has been shown to expand life expectancy (6.24 months) compared with single-agent gemcitabine (5.91 months), although its use is limited by the high frequency of *KRAS2* mutations in pancreatic cancers [142,144]. The lack of progress in treating pancreatic cancers with the limited success rate of adjuvant chemotherapy underscores the dire need for the discovery and development of new chemotherapeutic agents for this disease.

### 4.4. The Antimetabolite Class of hRR Inhibitors: Tezacitabine

Like gemcitabine, tezacitabine is a structural analog of deoxycytidine, although it is much less susceptible to metabolism by cytidine deaminase [145,146,147]. The diphosphate form irreversibly inhibits the hRRM1 subunit and incorporation of the triphosphate form into DNA strands, leading to strand breaks and apoptosis [145,148,149,150,151,152,153]. Clinical evaluation of tezacitabine has thus far included phase I and II studies of solid refractory tumors as a monotherapy and in combination with cisplatin for the treatment of gastrointestinal cancer and in combination with 5-fluorouracil for the treatment of advanced solid tumors [147,151,154,155,156]. Trials of both the monotherapy and combination regimens have not progressed beyond phase II due to a lack of efficacy.

### 4.5. Nucleoside Analog DMDC

Like gemcitabine and tezacitabine, 2′-deoxy-2′-methylidenecytidine (DMDC) is a structural mimic of deoxycytidine that is activated by intracellular phosphorylation to the diphosphate and triphosphate forms [157]. The diphosphate inhibits the hRRM1 subunit, while the triphosphate inhibits DNA polymerases, leading to DNA strand breaks and apoptosis [158]. DMDC is resistant to metabolism by cytidine deaminase and was proven effective in xenograft models of cancers, where cytidine deaminase activity is high and gemcitabine response is typically low [159,160,161]. Phase I trials of DMDC indicated a high rate of severe hematological toxicities that terminated its development as a chemotherapeutic [160,162,163].

### 4.6. hRRM1 Covalent Modifier Caracemide

The hydroxylamine derivative caracemide was demonstrated to irreversibly inhibit active site cysteine residue in *E. coli* RR1 [164]. Interestingly, although no direct effects on the RR2 subunit are observed, the holocomplex was found to be over 30 times more sensitive to inhibition by caracemide than isolated RR1. Although caracemide demonstrated promising antiproliferative effects in lymphocytic leukemia and Ehrlich ascites carcinoma cells, its efficacy is severely limited by its instability in human plasma and the resulting neurotoxicity of its metabolites [165,166,167]. Caracemide was evaluated in a wide array of phase I trials as an anticancer agent but rarely progressed to phase II due to severe dose-limiting toxicities, including pain, renal failure, respiratory failure, and neurological dysfunction [168,169,170,171,172,173].

## 5. Non-Nucleoside Inhibitors of hRRM2

To overcome the limitations of the antimetabolite inhibitors of hRRM1, there were attempts to develop non-nucleoside inhibitors that target the hRRM2 subunit or the hRRM1–hRRM2 interface. In this section, we discuss a broad selection of the most common classes of clinically evaluated hRRM2 inhibitors: radical scavengers, metal chelators, and compounds and biologics that disrupt binding between the hRRM1 and hRRM2 subunits.

### 5.1. The Radical Scavengers: Hydroxyurea

The most important of the radical scavenger class is hydroxyurea, which was one of the first anticancer therapeutics identified in 1953. Hydroxyurea quenches the tyrosyl radical of hRRM2, preventing catalysis of the hRR holocomplex [88,89]. Hydroxyurea has been used clinically for decades to treat a variety of diseases, including chronic myeloid leukemia (CML), polycythemia vera, and cervical cancer, but its use is limited by its poor efficacy, inconvenient dosing schedule, and a high rate of natural resistance in patients [174,175,176,177,178,179,180,181]. Currently, hydroxyurea is more commonly administered as a combination therapy, where it was evaluated in clinical trials for malignant gliomas (with imatinib) [182], glioblastomas (with cisplatin and ara-C or with imatinib) [183,184], and squamous cell carcinoma of the head and neck (with radiation and 5-fluorouracil) [185]. As a monotherapy, hydroxyurea is FDA-approved for the treatment of myeloproliferative disorders and sickle cell disease [186,187]. 

### 5.2. Metal Chelators: Triapine, Didox, Trimidox, Desferrioxamine, and Gallium Complexes

Triapine was evaluated as an anticancer therapeutic in phase I and phase II clinical trials and inhibits hRR by chelating Fe^III^ from the radical center in hRRM2 [188]. A member of the heterocyclic carboxyaldehyde thiosemicarbazones (HCTs), triapine coordinates iron in a 2:1 ligand-to-iron ratio through a N*-N*-S* tridentate system, forming an octahedral complex [90,91,92]. In hRR inhibition, the triapine–Fe^III^ complex is quickly reduced to a more favorable triapine–Fe^II^ complex, which was shown to activate molecular oxygen to a ROS, quenching the essential tyrosyl radical of hRRM2, [189]. Triapine was also shown to degrade DNA in the presence of hydrogen peroxide and iron, suggesting that this drug is not only an inhibitor of DNA repair/DNA synthesis but also a DNA-damaging agent [190]. Triapine was evaluated in several phase I and II studies for the treatment of leukemia, advanced solid tumors, advanced hematologic malignancies, head and neck cancer, and renal cell carcinoma [191,192,193,194]. In most cases, the response rate to monotherapy triapine was limited (0–7%) and high rates of grade 3 and 4 neutropenias were reported, leading to the termination of the studies [195]. Triapine was also evaluated in combination with various chemotherapies, including fludarabine for refractory acute leukemia and myeloproliferative disorder [191], doxorubicin for advanced solid tumors [196], and gemcitabine for non-small-cell lung cancer and advanced solid tumors [188,197,198,199]. While the combination with gemcitabine was discontinued due to high toxicity, results from a phase II study of combinations with fludarabine for refractory acute leukemia and myeloproliferative disorder were considered promising [200].

The hydroxyl-benzohydroxamic acid derivatives didox and trimidox are radical chelators that complex with Fe to inhibit the hRRM2 subunit [201,202,203,204,205,206,207]. These compounds are known to impair proliferation in a variety of cancer cell lines as monotherapies. Both compounds also showed synergistic effects with approved chemotherapies, both in vitro and in vivo [208,209]. When combined with either carmustine or temozolimide, didox worked synergistically to impair proliferation in brain cancer cell lines [208,209,210]. Didox further reduces tumor growth in Epstein–Barr-virus-positive nasopharyngeal carcinoma xenografts when combined with cidofovir [211]. Phase 1 and 2 studies of didox for various indications were completed, although no study has progressed beyond phase 2 [212,213,214,215]. While didox showed tolerable dosing and toxicity, its efficacy was generally not sufficient to warrant phase 3 evaluation. Didox has not been evaluated in clinical trials as a combination therapy. Trimidox has not been clinically evaluated but has demonstrated synergistic effects with tiazofurin, adriamycin, streptozotocin, ara-C, cisplatin, and cyclophosphamine in leukemia models [204,205,206,216,217].

Desferrioxamine (DFO), also known as deferoxamine (DFOA), is a well-characterized iron and aluminum chelator that is commonly used to treat iron overdose, hemochromatosis, and aluminum toxicity in dialysis patients. DFO is also capable of inhibiting hRR activity by chelating Fe^III^, forming a 1:1 ligand–metal complex [218]. While DFO does not directly affect the hRRM2 subunit, sequestration of intracellular iron prevents the activation and regeneration of the tyrosyl radical center, thus inhibiting hRR activity [219,220,221]. This inhibition is reversible with the addition of iron and appears to affect p53R2 more potently than hRRM2. DFO showed antiproliferative effects in leukemia and neuroblastoma cells in vitro and in vivo [222,223,224,225]. Clinical trials in neuroblastoma showed some efficacy, but the use of DFO as an antiproliferative agent is limited by its poor cell permeability and very short half-life [222,223,226,227,228]. An additional limitation is the ability of DFO to induce overexpression of hypoxia-inducible factor 1α (HIF1α); a recent report by Lang et al. indicated that co-administration of DFO and the HIF1α inhibitor lificiguat synergistically impaired proliferation in pancreatic cancer cell lines [229]. Encapsulation of DFO in transferrin receptor 1 targeting lysosomes improved cellular uptake and prolonged circulation time in vivo, suggesting that the clinical limitations of DFO could be overcome with novel delivery strategies. 

The gallium variants gallium nitrate and gallium maltolate have been clinically evaluated as therapeutics for a wide variety of cancers since the 1980s; in 1991, gallium nitrate was approved for cancer-related hypercalcemia [230]. Gallium mimics ferric iron and is taken up by rapidly proliferating cells, which need iron for DNA synthesis. However, gallium inhibits RR and hence inhibits DNA synthesis, triggering apoptosis [231,232,233]. A recent study by Chitambar et al. found that gallium maltolate treatment of glioblastoma and glioblastoma stem cells led to a loss of mitochondrial reserve capacity which was followed by decreased oxygen consumption and decreased activity of hRRM2, which requires iron for the catalytic ferric-tyrosyl radical cluster [234]. In this way, gallium formulations that interfere with iron metabolism may also indirectly inhibit hRRM2.

Motexafin gadolinium is a synthetic metallotexaphyrin that inhibits both hRR and thioredoxin reductase [235]. Its hRR inhibition is via the replacement of Fe^III^ iron in hRRM2. Motexafin gadolinium accumulates in tumor cells preferentially due to their increased metabolism and generates ROS, resulting in apoptosis. It was evaluated in numerous phase 1 and 2 clinical trials as both a monotherapy and combination therapy for non-small-cell lung cancer [236,237], non-Hodgkin’s lymphoma [238], glioblastoma and other brain cancers [239,240,241,242,243,244], chronic lymphatic leukemia [245], and renal cancer [246]. A phase 3 study of motexafin gadolinium in non-small-cell lung cancer with brain metastasis led to increased disease progression from 10 months to 15 months and suggested improvements to memory and executive function in patients [243]. The drug remains active in phase 3 trials for various other cancers.

### 5.3. A Disruptor of hRRM1–hRRM2 Binding: COH29, Antisense Oligonucleotides, and siRNAs

The lack of a fully ordered α_2_β_2_ complex structure for hRR has limited the options to develop small molecule interference disruptors as a known structure is a prerequisite for knowledge-based drug design. Therefore, most drug design attempts were made with either the hRRM1 structure or the hRRM2 structure. *In silico* modeling by the Yen group targeting the p53R2 structure identified the non-nucleoside molecule COH29 [247]. Moreover, COH29 inhibits PARP P, suggesting the involvement of polypharmacology [248]. This compound is expected to bind a small pocket on the C-terminal tail of hRRM2 away from the diferric-tyrosyl radical cluster. COH29 reduced proliferation in most of the NIH 60 panel of cancer cell lines and induced S-phase arrest. Twice-daily oral dosing of COH29 (50 and 100 mg/kg) was further found to impair tumor growth and prolong survival in mice with MOLT-4 acute myeloid leukemia or TOV11D ovarian cancer xenografts. In BRCA1-deficient human breast cancer cells, COH29 was found to impair growth by interfering with DNA repair pathways [248]. The drug is currently undergoing phase 2 clinical evaluation.

Additional biologics were developed to inhibit the hRRM2 subunit. GTI-2040, which is an antisense oligonucleotide targeting the hRRM2 mRNA, showed strong inhibition of many cancer types in vitro and in vivo and was studied clinically as both a monotherapy and a combination therapy for renal, prostate, AML, NSCL, and metastatic breast cancers [249,250]. However, GTI-2040 has never progressed beyond phase 2 trials due to limited efficacy. An additional antisense oligonucleotide, namely, GTI-2501, targets the hRRM1 subunit and is currently under evaluation in phase 2 trials [251]. GTI-2501 was shown to decrease mRNA and protein levels of hRRM1 in a variety of tumors and impair tumor progression in vivo [77,252]. A clinical safety study of CALAA-01 (NCT00689065) was terminated for unknown reasons. 

Non-nucleoside inhibitors of hRR showed promise in clinical trials as chemotherapeutics for a wide range of cancers. As such, interest remains high in identifying novel non-nucleoside inhibitors of hRR as leads for drug development. In the remainder of the review, we discuss emerging efforts to identify non-nucleoside inhibitors targeting hRR.

## 6. Continuing Efforts to Identify Novel Inhibitors of hRR

Efforts to develop novel inhibitors of hRR remain very active and have employed a variety of strategies to target either the hRRM1 or hRRM2 subunit. In this section, we review some of the novel classes and methods of hRR inhibition that have not yet achieved clinical evaluation.

### 6.1. Peptides Targeting the hRRM1 Subunit

There have been multiple efforts to target the RR1 subunit with peptides that can compete with the RR2 subunit for binding [253,254,255,256,257,258,259]. The first such study was reported in 1986 and demonstrated that the nonapeptide YAGAVVNDL derived from the herpes simplex virus R2 (HSV-RR2) could inhibit HSV-RR [253,254]. Parallel studies established that the majority of the binding affinity could be attributed to the pentapeptide VVNDL, which led to a series of structure–function studies that identified pentapeptides that targeted HSV-RR1 with nanomolar binding affinities.

**Figure 4:**
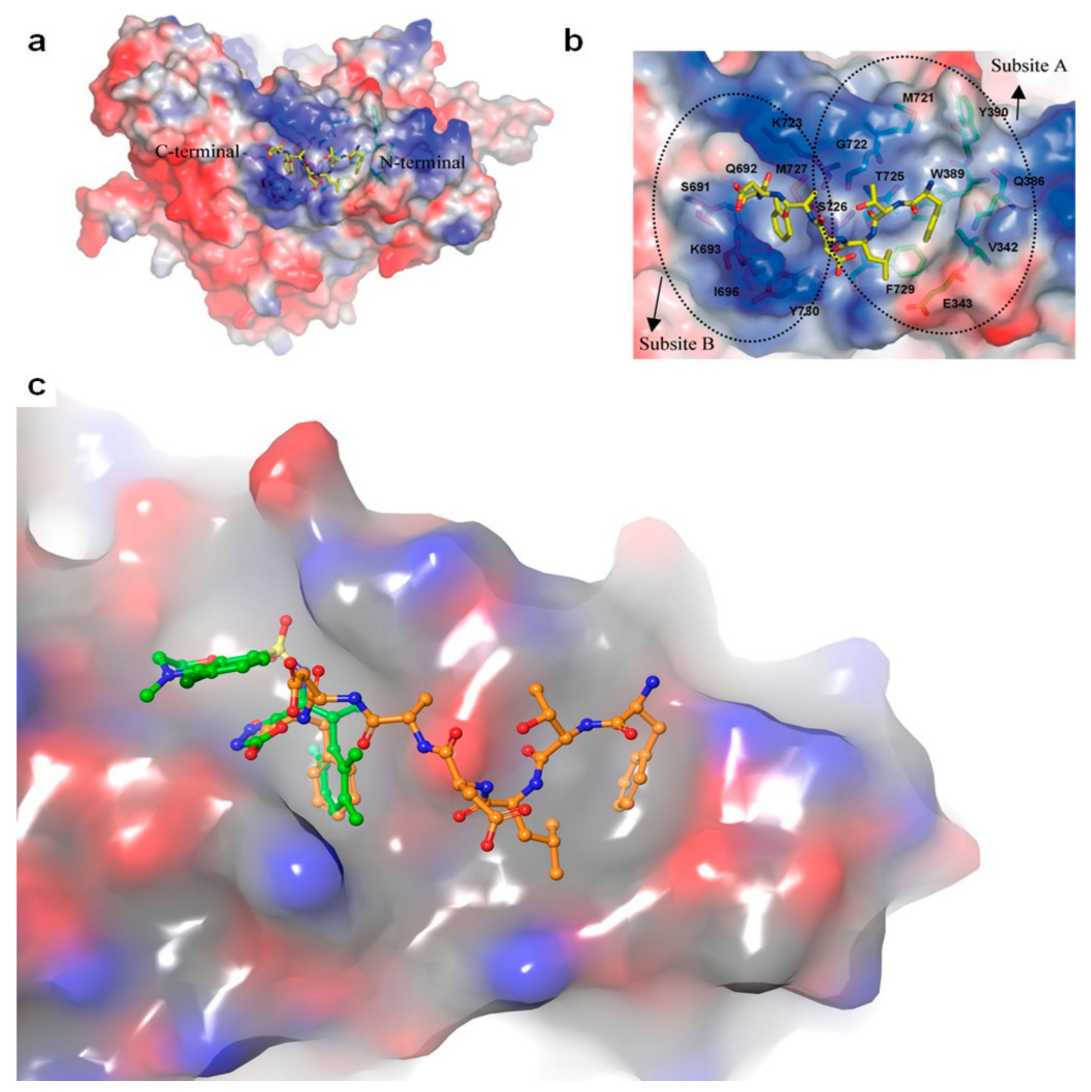
Structure of the ScR1-P7 complex. (**a**) The electrostatic potential surface showing the R2 binding site on R1. P7 is shown in yellow. (**b**) The electrostatic potential surface of the P7 binding site, with subsites (**a**,**b**) clearly indicated. (**c**) Superposition of P7 (orange) with small molecule TAS1553 (green). TAS1553 occupies subsite B of the peptide binding region. Figure adapted with permission from Xu et al. [118].

An *N*-acetyl heptapeptide derived from the C-terminus of the mouse and human mRR2 and hRRM2 subunits named P7 (*N*-Ac-FTLDADF) was found to inhibit mammalian RR with an IC_50_ of ~20 µM [258]. This peptide and its derivatives are stronger inhibitors of the α_2_β_2_ dimer complex than the α_6_β_n_ complex. Crystal structures of P7 and its derivative P6 in complex with *Saccharomyces cerevisiae* RR1 identified two contiguous binding sites that are highly conserved amongst eukaryotic RR1s (Figure 4) [260]. Subsite A was found to consist of highly hydrophobic residues that housed the N-terminal residues of the peptides, while subsite B carried a highly positively charged surface that favorably interacted with the negatively charged carboxylate terminals. These structural studies support the experimental trend of maximal potency occurring at peptide lengths of at least seven residues. However, a recent crystal structure of a non-peptidyl small molecule inhibitor TAS1553 bound to the hydrophobic site of P7 (PDB ID: 6L3R) was reported, demonstrating that small molecules can bind at the site and can be developed as cancer therapies.

### 6.2. Alkoxyphenols Targeting the hRR2 Subunit

Alkoxyphenols were demonstrated to quench the tyrosyl radical of the hRR2 subunit of mouse and herpes simplex virus 2 via a radial redox reaction [261,262]. p-Alkoxyphenols were shown to inhibit RR in Novikoff hepatoma, leukemia, and melanoma cells to impair proliferation [263]. Thus far, these compounds have not advanced to clinical evaluation.

### 6.3. Bivalent Inhibitors of hRR

Several attempts to develop bivalent inhibitors targeting hRR were undertaken. In 2000, Wu et al. reported the development of a bivalent inhibitor of mouse RR that was designed to target the C-site and S-site of the mRR1 subunit [264]. The compound features ADP and dGDP linked by a 1,6-hexane-(bisethylenesulfone) tether. Despite a promising binding affinity of 12 µM, this compound was found to bind only the S-site. In 2014, Petrelli et al. reported the design of bivalent inhibitors linking 3′-*C*-methyladenosine and valproic acid, which are known inhibitors of RR and histone deacetylase (HDAC), respectively [265]. While the bivalent compounds remained active inhibitors of RR, they were unable to target HDAC. One analog, namely, A167, demonstrated potent antiproliferative effects in a broad variety of cancer cell models and reduce dNTP pools in HL-60 cells. A167 was found to compete with ATP at the A-site to inhibit the hRRM1 subunit.

### 6.4. S-Site Inhibition by the Non-Natural Nucleotide 5-NITP

In 2012, Mohammed et al. evaluated the ability of a set of ten 5-substituted indolyl-2′-deoxynucleoside triphosphates (5-NIdR) to inhibit hRRM1 [266]. The 5-NIdR scaffold was selected for evaluation due to its ability to mimic the size and shape of the allosteric inhibitor dATP. One such compound, namely, 5-NITP, was found to inhibit hRR with an IC_50_ of 170 µM and a *K*_d_ of 44 µM. Interestingly no inhibition was observed when CDP was used as the substrate, suggesting that the compound could bind to the S-site as opposed to the C-site. This theory was supported by gel filtration studies, which indicated that the predominant oligomeric form of the hRRM1-5-NITP complex was the dimer complex as opposed to the hexamer complex. A crystal structure of 5-NITP in complex with the *Saccharomyces cerevisiae *RR1 subunit confirmed that 5-NITP binds to the S-site preferentially and induced cytotoxicity in human leukemia cells (PDB ID: 3RSR). 5-NITP was further demonstrated to induce cell-cycle arrest at the S-phase, consistent with the inhibition of DNA synthesis in human leukemia cells.

### 6.5. Resveratrol Analog 4,4′-Trans-Dihydroxystiblene (DHS)

The resveratrol analog 4,4′-trans-dihydroxystilbene (DHS) was known to impair the proliferation of a variety of cancer cells, including human promyelocytic leukemia, breast cancer, neuroblastoma, and lung cancer cells although its mechanism of action was unclear [267,268,269,270,271,272]. In 2018, Chen et al. demonstrated that DHS induced cell-cycle arrest and decreased dNTPs pool in a manner consistent with hRR inhibition [273]. Thermal shift assays identified the direct binding of DHS to the hRRM2 subunit and site-directed mutagenesis confirmed that Val 146, Ser 150, Gln 151, and Ile 166 are critical for DHS binding and inhibition. Chen et al. further identified that DHS induced hRRM2 degradation by the proteosome degradation pathway [273]. Xenograft models further established that DHS combination therapies were able to overcome gemcitabine resistance in pancreatic cancer models and cisplatin resistance in ovarian cancer models and delayed tumor progression [273].

### 6.6. Modulation of the hRRM1 Oligomeric Equilibrium by OxoIsoIndoLys

Initial attempts by Dealwis and colleagues to identify non-nucleoside inhibitors of hRR that could inactivate the enzyme by manipulating its tightly regulated oligomeric equilibrium were reported [274]. The prevailing model of allostery is that hRR is active as an α_2_β_2_ complex, but under physiological conditions, exists primarily as an active α_6_β_n_ hexamer (*n* = 2, 4, or 6) with a small population of dimers present [28,29]. hRR inhibitors, such as clofarabine and gemcitabine, were shown to induce inactive hRRM1 α_6_β_2_ hexamers, supporting the role of oligomeric modulation in the small molecule inhibition of hRR [83,119]. The central hypothesis of the study was that by targeting the hexamer interface with high throughput virtual screening, one could identify small molecules that bind to and stabilize the inactive hexamer conformation preferentially, thus shifting the natural oligomeric equilibrium toward the inactive state. Screening of the University of Cincinnati compound library (formerly the Proctor and Gamble library) identified 91 compounds that were expected to bind and inhibit hRRM1. Docking was directed toward the hexamer interface of the inactive dATP-bound hRRM1 hexamer model constructed from the *S. cerevisiae* dATP-induced hexamer (PDB: 3PAW) [30]. The docking site was defined as the N-terminal 16 residues from adjacent dimers. The top hits from the docking studies were subject to biophysical fluorescence quenching studies to determine dissociation constants against hRRM1. Out of the 91 candidates, 51 compounds were chosen to have greater than 20% fluorescence quenching and hence deemed as reasonable binders of hRRM1. These compounds were screened at two doses (10 and 1 µM) in multiple cancer cell lines to identify compounds with antiproliferative properties. The 10 most potent inhibitors were verified to inhibit hRRM1 and inhibit the enzymatic activity of recombinant hRR with micromolar potency [274].

A crystal structure of hRRM1 in a complex with a phthalimide-based inhibitor, namely, OxoIsoIndoLys, verified that this compound binds to the hexamer interface at the N-terminus and gel filtration studies demonstrated that this compound promoted hRRM1 hexamers. The structure of the hRRM1–OxoIsoIndoLys complex was determined down to a 3.7 Å resolution [274]. The compound was observed to form non-polar interactions with the N-terminus of one dimer unit, although a polar contact was observed with Ala 49. In the presence of 50 µM dATP, recombinant hRRM1 exists in equilibrium between α_2_ dimers and α_6_ hexamers, where the dimer: hexamer ratio is 1:3. Upon addition of 1 mM OxoIsoIndoLys, the dimer to hexamer ratio increased seven-fold, verifying that OxoIsoIndoLys can modulate the hRRM1 oligomeric equilibrium.

### 6.7. NSAH and Other Acylhydrazones Targeting the C-Site of hRRM1

**Figure 5:**
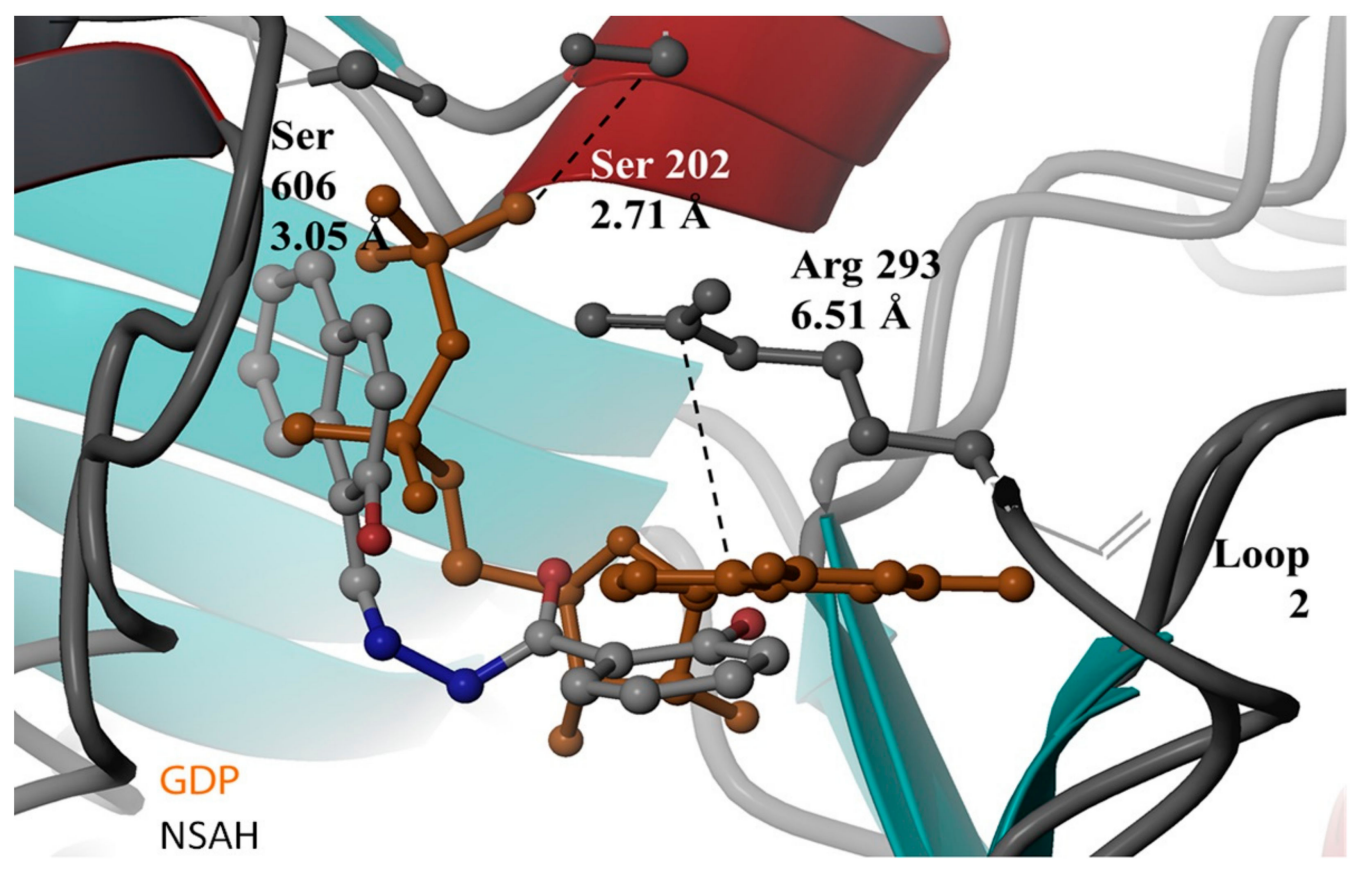
Crystal structure of NSAH at the C-site of hRRM1. NSAH is shown in gray, substrate GDP is shown in orange. Figure adapted with permission from Huff et al. [275].

In 2017, the Dealwis lab identified the first non-nucleoside small molecule capable of binding the C-site of hRRM1, which was a naphthyl salicylic acid hydrazone named NSAH [275]. In an effort to identify unique chemical scaffolds likely to bind the site, an *in silico* screen targeting the C-site was performed using Glide in the Schrödinger software suite. Cancer cell growth inhibition assays identified two hydrazone derivatives, namely, NSAH and a related naphthyl acyl hydrazone (NAH), with potent cellular activity (IC_50_ ~250 nM NSAH, 600 nM NAH) and cell-free inhibition against recombinant hRR (IC_50_ = 32 µM NSAH, 40 µM NAH). The direct binding of NSAH to recombinant hRRM1 was determined using a fluorescent quenching assay, establishing the *K*_D_ as 37 ± 4 µM. The enzymatic IC_50_ was established at 32 ± 10 µM. A series of kinetics experiments demonstrated that NSAH inhibited hRR reversibly and competitively. IC_50_ values of NSAH ranged from 220–500 nM in a broad panel of cancer cells, while no cytotoxicity was observed in normal human blood progenitor cells at concentrations up to 1 µM, indicating NSAH has a much broader therapeutic index than the C-site inhibitor gemcitabine; there are no viable cells at a gemcitabine concentration of 35 nM. A crystal structure of the dimeric form of hRRM1 bound to NSAH at a 2.66 Å resolution confirmed that NSAH binds to the C-site, occupying a similar position to the binding pocket normally observed for diphosphate substrate binding (PDB ID: 5TUS, Figure 5) [275]. NSAH can adopt either a cis or trans conformation, and the cis isomer was observed in the crystal structure. 

A series of structure–activity relationship studies were undertaken to optimize the potency of NSAH [276]. These efforts initially focused on modification of the substituent groups of the benzene and naphthalene rings while leaving the core scaffold intact. Notably, the synthetic scheme employed in these studies preferentially afforded *E*-isomers. This initial study identified that ortho-polar substitution to the benzene moiety of the hydrazone scaffold was required for hRR inhibition. An additional series of 13 hydrazones were then designed with modifications focused on disassociating the fused ring system into a biphenyl moiety with various substitutions [277]. All of the modified hydrazones were found to bind hRRM1 with lower *K*_D_ values than NSAH (*K*_D_ = 22.0 ± 5.67 µM), where 12 of the 13 NSAH analogs reported *K*_D_ values below 10 µM. Screening of the compounds in Panc1 cell lines identified three analogs with potent antiproliferative effects, TP 4, 5, and 6 (IC_50_s > 1.5-0.393 µM). In fact, TP 6 had a twofold lower IC_50_ compared with NSAH. Interestingly, all three of the most potent analogs contained a biaryl replacement of the naphthalene ring moiety from the parent NSAH structure while maintaining the phenol and hydrazone linker. Further, the biaryl replacement in all three analogs includes a common guaiacol methoxyphenol ring that maintains the polar hydrogen bond donor position of the parent naphthol moiety. The removal of this methoxy group in TP2 and -3 resulted in at least a twofold decrease in cellular potency and induced a different binding orientation when docked at the C-site. These data indicate that including an electronegative element in the biaryl ring system could further increase the potency.

### 6.8. A Novel High Throughput PCR Assay Identifies Chemically Distinct RR Inhibitors

In an effort to identify RR inhibitors with antiviral activity, the Tholander and Sjöberg groups developed a PCR-based method for screening RR activity in microplate format, allowing for a higher throughput assessment of potential inhibitors than traditional methods [278]. A screen of >1300 compounds against *P.aeruginosa* RR identified 27 compounds with reported IC_50_ values between 200 nM and 30 µM. Four of these compounds, namely, NSC36758, NSC45383, NSC361666, and NSC228155, demonstrated antibacterial properties against *P.aeruginosa*, and both NSC361666 and NSC228155 were shown to deplete dNTP pools, which is consistent with RR inhibition. Both NSC45383 and NSC228155 were found to quench the *P.aeruginosa* RR2 radical, although these effects could be due to either iron chelation or generation of reactive oxygen species [279]. A comparison of the high throughput assay hits with reported cytotoxicity data from the NCI-60 panel of cancer cell lines revealed a selection of unique hRR inhibitors with anticancer properties, including NSC73735 [280]. Further evaluation of NSC73735 indicated that it prevented α hexamerization, in contrast to other established hRRM1 inhibitors, such as clofarabine. The selective cytotoxicity observed toward leukemia cell lines makes NSC73735 an attractive compound for further development as an anticancer agent. The characteristic phenotype of hRR inhibition, namely, cell cycle arrest and depletion of dNTP pools, was observed in HL-60 cells after treatment with NSC73735. Interestingly, these effects were reversible, in contrast to the less selective hRR inhibitor hydroxyurea. However, this compound is also known to inhibit dihydroorotate dehydrogenase and interfere with the synthesis of pyrimidine nucleotides [281,282]; it is possible that the depletion of dNTP pools can be promoted by both mechanisms.

### 6.9. Transmetalative Iron Chelators That Inhibit hRRM2

In 2021, Gaur et al. reported an iron chelator transmetalative approach to inhibit the hRRM2 subunit by exchanging a titanium(IV) ion for Fe(III) and depleting the intracellular iron pool [283]. A series of chemical transferrin mimetics (cTfms) were evaluated as the metal chelator due to their thermodynamic preference for binding Fe(III) over Ti(IV), thereby driving the intracellular transmetalation. The hexadentate chelator* N,N’*-di(*o*-hydroxybenzyl)-ethylenediamine-*N,N’*-diacetic acid (HBED) and the FDA-approved tridentate chelator Deferasirox both exhibited potent cytotoxicity to Jurkat cells by decreasing intracellular iron. Both the Ti(HBED) and Ti(Deferasirox)_2_ complexes were found to alter the nucleotide substrate pool and induce cell-cycle arrest consistent with RR inhibition.

## 7. Conclusions and Perspectives

Given the number of clinically approved cancer chemotherapeutics that target either the hRRM1 or hRRM2 subunit of ribonucleotide reductase, hRR remains a compelling target for cancer chemotherapy. Currently, hydroxyurea, which targets hRRM2, and the nucleoside analogs that target hRRM1 are the only clinically approved drugs for the treatment of cancer. While most nucleoside analogs were approved for the treatment of leukemia, gemcitabine has demonstrated efficacy against solid tumors and is the most widely used hRR targeting chemotherapy. However, nucleoside analogs suffer from dose-limiting toxicity (possibly due to their chain-terminating activity against the DNA polymerases) and narrow therapeutic windows. Moreover, chemoresistance develops after a few months of treatment, rendering the drug ineffective. Small subunit targeting drugs, such as hydroxyurea, that interfere with the di-iron cluster through scavenging and chelating activity have their own drawbacks due to a lack of target specificity and lack of efficacy. Despite the limitations of these chemotherapies, new on-target therapies, such as kinases and phosphatase inhibitors, have limited use in cancers such as pancreatic cancer, triple-negative breast cancer, and glioblastomas. Therefore, it will be useful to develop new functional “gemcitabine-like” molecules that will maintain their efficacy but lack their current deficiencies. In order to overcome the aforementioned limitations of current hRR drugs, novel strategies were developed that include non-nucleoside small molecule reversible competitive inhibitors that may function more effectively than gemcitabine and other nucleosides and to discover subunit and hexamer interface blocking inhibitors that will not bind at orthostatic sites and hence possess on-target specificity. Future clinical trials will demonstrate whether these new strategies will yield promising therapeutics for the treatment of cancer. After decades of research, RR continues to be a rich and complex target for antiproliferative diseases.

## Figures and Tables

**Table 1:** FDA-approved hRR inhibitors and their anticancer applications.

Structure	Name	Cellular Mechanism	RR Subunit Targeted	Mechanism of RR Inhibition	Indications: Single Agent	Indications: Combination
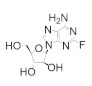	Fludarabine	Interferes with DNA synthesis and repair mechanisms	hRRM1	Induces formation of inactive a_6_ hexamers	Chronic lymphocytic leukemia	With cyclophosphamide, mitoxantrone, dexamethasone, and rituximab: non-Hodgkin’s lymphoma With cyclophosphamide, mitoxantrone, dexamethasone, and granulocyte colony-stimulating factor: acute myeloid leukemia
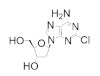	Cladribine	Interferes with DNA synthesis and repair mechanisms	hRRM1	Induces formation of inactive a_6_ hexamers	Hairy cell leukemia B-cell chronic lymphocytic leukemic	
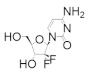	Gemcitabine	Interferes with DNA synthesis and repair mechanisms	hRRM1	Alkylates the C-site irreversibly	Pancreatic cancer Off label: cholangiocarcinoma and other biliary tract cancers	With nab-paclitaxel: pancreatic cancer With cisplatin: advanced or metastatic bladder cancer; advanced or metastatic non-small cell lung cancer With carboplatin: ovarian cancer With paclitaxel: metastatic breast cancer
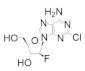	Clofarabine	Interferes with DNA synthesis and repair mechanisms	hRRM1	Induces formation of inactive a_6_ hexamers	Relapsed or refractory acute lymphoblastic leukemia	
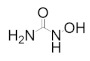	Hydroxyurea	Radical Scavenger	hRRM2	Quenches the radical center	Chronic myelogenous leukemia	

**Table 2:** Structure and mechanism of hRR inhibitors.

Structure	Name	Cellular Mechanism	RR Subunit Targeted	Mechanism of RR Inhibition
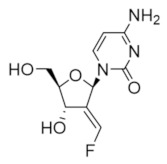	Tezacitabine	Interferes with DNA synthesis and repair mechanisms	hRRM1	Irreversibly modifies the C-site
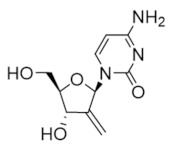	2′-deoxy-2′-methylenecytidine (DMDC)	Interferes with DNA synthesis and repair mechanisms	hRRM1	Inhibits the C-site
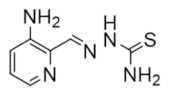	Triapine	Metal chelation	hRRM2	Chelates Fe^III^ from the radical center
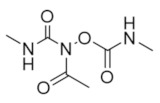	Caracemide	Radical Scavenger	hRRM1	Quenches the radical center
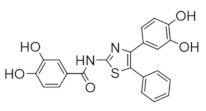	COH29	Interferes with DNA synthesis and repair mechanisms	hRRM2	Binds hRRM2 near the C-terminal tail to prevent association with hRRM1
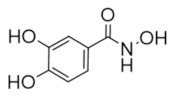	Didox	Radical Scavenger	hRRM2	Chelates Fe^III^ from the radical center
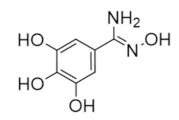	Trimidox	Radical Scavenger	hRRM2	Chelates Fe^III^ from the radical center
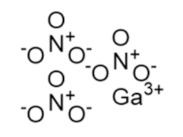	Gallium nitrate	Fe^III^ mimic	hRRM2	Interferes with Fe^III^ radical center
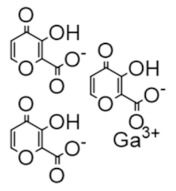	Gallium maltolate	Fe^III^ mimic	hRRM2	Interferes with Fe^III^ radical center
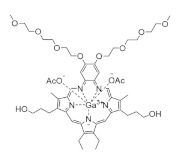	Motexafin gadolinium	Fe^III^ mimic	hRRM2	Interferes with Fe^III^ radical center
	GTI-2040	antisense ologonucleotide	hRRM2	Inhibits hRRM2
	GTI-2501	antisense ologonucleotide	hRRM2	Inhibits hRRM2
	CALAA-01	siRNA-containing nanoparticle	hRRM2	Inhibits hRRM2

**Table 3:** Structure and mechanism of hRR subunit inhibitors.

Structure	Name	RR Subunit Targeted	Mechanism of RR Inhibition
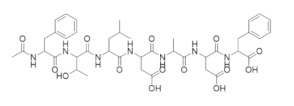	P6	RR1	Prevents binding of the RR2 subunit
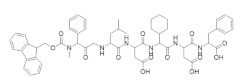	P7	RR1	Prevents binding of the RR2 subunit
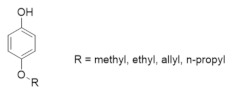	p-Alkoxyphenols	RR2	Quenches the tyrosyl radical
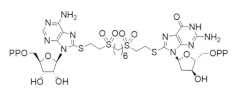	ADP-S-HBES-S-dGTP	RR1	Targets the S-site
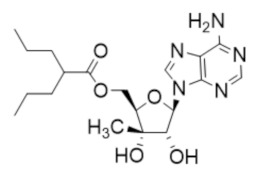	A167	RR1	Compeites with ATP to bind the A-site
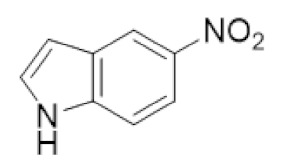	5-NITP	RR1	S-site inhibitor that prevents RR1 hexamerization
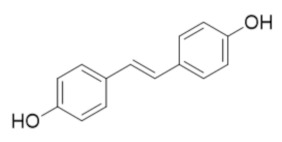	DHS	RR2	Induces degradation of RR2 proteosome degredation pathway
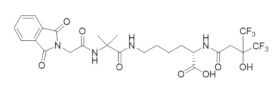	OxolsolndoLys	RR1	Induces formation of inactive a_6_ hexamers
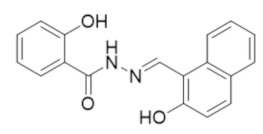	NSAH	RR1	Reversible C-site inhibitor
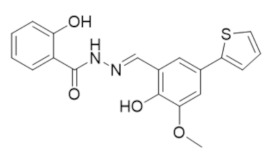	TP6	RR1	Reversible C-site inhibitor
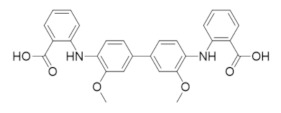	NSC73735	RR1 and RR2	Prevents alpha subunit hexamerization and quenches the tyrosyl radical of RR2
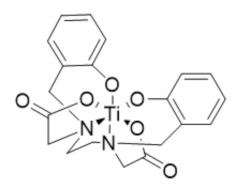	Ti(HBED)	RR2	Depletes intracellular labile iron pools by transmetalation
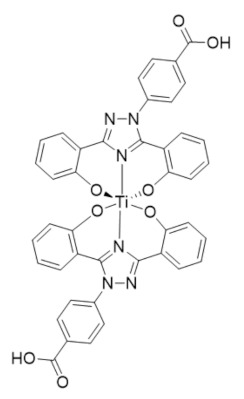	Ti(Deferasirox)_2_	RR2	Depletes intracellular labile iron pools by transmetalation
